# Barriers to Physical Activity in a Population-based Sample of Children and Adolescents in Isfahan, Iran

**Published:** 2010

**Authors:** Roya Kelishadi, Shohreh Ghatrehsamani, Mohsen Hosseini, Parisa Mirmoghtadaee, Samaneh Mansouri, Parinaz Poursafa

**Affiliations:** 1 Professor of Pediatrics, Departmentof Pediatric Preventive Cardiology, Isfahan Cardiovascular Research Center, Isfahan University of Medical Sciences (IUMS), Isfahan, Iran; 2 Research Assistant, Department of Pediatric Preventive Cardiology, Isfahan Cardiovascular Research Center, IUMS, Isfahan, Iran; 3 Assistant Professor of Biostatistics, Department of Biostatistics& Epidemiology, School of Public Health, IUMS, Isfahan, Iran; 4 Specialist of Community Medicine, School of Pharmacy, IUMS, Isfahan, Iran; 5 Biostatistician, Department of Biostatistics& Epidemiology, School of Public Health, IUMS, Isfahan, Iran

**Keywords:** Physical activity, Pediatric, Barriers, Prevention, Iran

## Abstract

**Objectives:**

This study was conducted to explore the barriers to physical activity in a representative sample of Iranian children and adolescents.

**Methods:**

The study was conducted in 2007 in urban and rural areas of Isfahan district in Iran. In the qualitative part, we used the grounded theory approach, including semi-structured focus group discussions and indepth interviews. The quantitative part comprised 600 randomly selected students.

**Results:**

The qualitative study included 34 school students (16 girls), 20 parents (11 mothers) and 11 school staff. All students disclosed that studying was a priority. They pointed to lack of safe and easy-access place for physical activity and unsupportive family as the main barriers. Lack of self-confidence and low selfworth were the two other concepts developed in this context. Parents pointed to lack of safe and easy-access place for activity followed by the priority of studying. The concepts derived from interviews with school staff included unhealthy modeling of parents, priority of studying, and inadequate public knowledge about how to integrate physical activity in routine daily life. The quantitative survey comprised 600 students including 286 (47.8%) girls. Parents’ education level had inverse association with children’s physical activity level. Significant inverse associations of self-efficacy and physical activity levels were documented.

**Conclusions:**

Increasing the public knowledge about adopting physical activity habits in routine daily life, informing the families and students about the benefits of physical activity to improve learning, as well as providing safe places such as using the school facilities in non-school hours should be considered in planning effective preventive strategies and interventions.

## INTRODUCTION

Physical activity has several physical, psychological and social benefits for all age groups including children and adolescents.^1^ Moreover, it is suggested that the physical activity habits in adulthood origins from childhood.^2^ Physical inactivity contributes to the increasing health burden of obesity and type 2 diabetes among youths.^1^,^3^–^5^ Used as one of the strategies for childhood health promotion, the physical exercise also plays an important role in obesity prevention.^6^ Several school-based and community-based programs have been developed to increase awareness among teachers, parents and children about the health benefits of an active lifestyle.

However, increase in knowledge does not necessarily result in increase in physical activity level. Hence, it is important to understand the determinants of physical inactivity among children and adolescents to design proper interventions to increase physical activity in different populations. A large body of evidence indicates that socioeconomic inequalities and environmental characteristics have profound effects on health status and health behaviors.^7^ Age, gender and race are associated with physical inactivity; e.g., with increasing age, physical activity declines more rapidly among girls than among boys.^8^ The existing studies on social and environmental determinants of physical activity behavior are limited to Western countries.^9^–^15^ Three prominent theoretical models employed to study physical activity determinants among youth are the theory of research action (TRA)^1^, the theory of planned behavior (TPB)^17^ and sociocognitive theory (SCT).^18^ Socialcognitive factors such as attitudes, subjective norms, perceived behavioral control, and self-efficacy influence the decision to become physically active among youths.^19^ In this study, we explored the socio-economic and social-cognitive determinants of physical activity in a representative sample of children and adolescents in urban and rural areas of Isfahan district, Iran to find the barriers of promoting physical activity.

## METHODS

We used a combination of quantitative and qualitative research to provide a reasonable basis for the development of evidence-based strategies. The survey was conducted in 2007 by the Department of Pediatric Preventive Cardiology, Isfahan Cardiovascular Research Center affiliated to Isfahan University of Medical Sciences. After necessary accommodations with authorities of the Provincial Education and Training Organization, interviewers trained for this study were referred to elementary, middle and high schools of Isfahan, the second large city in Iran. Written informed consent was obtained from parents and oral assent from students. The study had a qualitative and a quantitative part.

### Qualitative study

A grounded theory approach was used for analyzing the participants’ experiences, and their perceptions. A total of 7 focus group discussions and 15 indepth interviews were conducted to explore the perceptions of students, parents and school staff about barriers to an active lifestyle of children and adolescents. Interviews and focus group discussions were conducted using a semi-structured interview conductor. The interview guide comprised openended questions to allow respondents to explain their own opinions and perceptions. The audiotaped records were transcribed verbatim and analyzed consecutively. Purposive sampling was used and followed with theoretical sampling.

### Quantitative study

The quantitative part comprised 600 students, aged 8-18 years, selected proportional to the population distribution in Isfahan district, i.e., 70% from urban and 30% from rural areas. They were selected by multistage random cluster sampling from different parts of the district with various socio-economic situations. Three types of questionnaires were used in this study: the questionnaire that evaluated the social-cognitive factors, the questionnaire that evaluated the level of physical activity and the questionnaire about socio-demographic variables such as age, gender, family income, household number, parents’ education and job. The first questionnaire was designed to measure attitudes, subjective norms, perceived behavioral control and self-efficacy about physical activity. The attitude questionnaire included 22 items that consisted of beliefs about the consequences of being physically active and a corresponding positive or negative evaluation of the consequences. The belief statements were rated on a 5-point Likert-type scale anchored by 1 (Disagree a lot) and 5 (Agree a lot); value statements were rated on a 5-point scale ranging from 1 (Very bad) to 5 (Very good). The attitude items were formed as a product of the belief and corresponding value item scores. The subjective norm questionnaire included 8 items that consisted of normative beliefs about the expectations of others towards being physically active and the corresponding motivation along with the expectations. The items were rated on a 5- point scale anchored by 1 (Disagree a lot) and 5 (Agree a lot). The subjective norm item scores were formed as a product of the normative belief and motive to comply item scores. The perceived behavioral control questionnaire included 4 items that pertained to perceptions of the ease/difficulty of being physically active. The items were rated on a 5-point scale anchors were 1 (Very easy/Agree a lot) and 5 (Very difficult/Disagree a lot). The self-efficacy questionnaire included 15 items that pertained to confidence in one’s ability to be physically active. The items were rated on a 5-point scale ranging from 1 (Very easy or Disagree a lot) to 5 (Very difficult or Agree a lot). This questionnaire was produced^19^ and validated previously,^20^ and in the current study we confirmed its validity and reliability in Iranian population. Physical activity level was assessed using short forms of the international physical activity questionnaire (IPAQ) with physical activity level categorized as low, moderate and vigorous levels on the basis of the IPAQ guidelines for data processing and analysis.^21^

### Statistical analysis

For the qualitative part of the study, data collection and analysis were done simultaneously according to the Grounded theory approach. The data obtained from interviews and focus group discussions were analyzed manually and were guided by constant comparative analysis.^22^ We used analytical tools, including asking questions and making comparisons, to find the properties of each concept. Interviewing was stopped when data saturation occurred. In the quantitative study, the dependent variable was the level of physical activity (low, moderate and vigorous) and the independent variables were social-cognitive variables, gender, parents’ education and job, number of household members and socioeconomic variables. The statistical analysis was done by SPSS for Windows software (SPSS Inc., Chicago, IL, USA; version 15.0) by using Chisquare, logistic regression analysis, Spearman correlation and analysis of variance statistical tests. The significance level was set at p value of less than 0.05.

## RESULTS

The participants of the qualitative study were 34 school students (16 girls and 18 boys), aged 8-18 years, 20 parents (11 mothers and 9 fathers)and 11 school staff. Irrespective of their school levels, all students disclosed that studying was a priority and mentioned that most of their time during the day was set to do their school-work. Moreover, they pointed to lack of safe and easy-access place for physical activity and group sport participation. They mentioned an unsupportive family as the main barrier for their daily physical activities. All girls and those boys with low self-esteem were less likely to make communication with peers and appear in group activities. Lack of self-confidence and low self-worth were the two concepts developed in this context. Parents pointed to lack of safe and easy-access place for physical activity of their children as the main barrier, followed by the priority of studying. The concepts derived from interviews with school staff included unhealthy modeling of parents, priority of studying, and inadequate public knowledge about how to make physical activity as part of routine activities of daily life. The quantitative survey comprised 600 students including 286 (47.8%) girls and 314 (52.2%) boys. In all school levels, the physical activity level was significantly higher in boys than in girls (p<0.001). As presented in Table 1, some indicators of low socio-economic status, as lower education of mothers and higher number of household members increased the physical activity of children and adolescents. Comparison of different levels of physical activity of children and adolescents according to the education level of their parents confirmed the inverse association of parents’ education level and children’s physical activity level (Table 2). The correlations of social-cognitive variables with different levels of physical activity are presented in Table 3. It shows significant inverse association of all variables studied, i.e., positive attitude, subjective norm, perceived behavioral control and self-efficacy with low physical activity level.

**Table 1:** Logistic regression analysis of socio-demographic variables with physical activity level in children and adolescents

Socio-demographic variables	Physical activity in children and adolescents	
	Odds ratio(95%CI interval)	P value	
**Father’s educational level**		
High school graduated		
Illiterate	0.53(0.11, 2.5)	0.43
Primary school	1.28(0.62, 2.67)	0.51
Less than high school	1.12(0.64, 1.97)	0.68
Some college	1.18(0.59, 2.35)	0.63
College graduated	1.21(0.51, 2.88)	0.67
University graduated	1.08(0.35, 3.32)	0.89
**Mother’s educational level**		
High school graduated		
Illiterate	11.22(1.43, 38.29)	0.02
Primary school	1.54(0.79, 2.97)	0.20
Less than high school	1.16(0.67, 2.00)	0.59
Some college	1.41(0.61, 3.27)	0.42
College graduated	1.21(0.51, 2.88)	0.67
University graduated	0.32(0.01, 7.24)	0.48
**Father’s occupation**		
Worker		
Employee	1.98(0.97, 4.06)	0.06
Faculty member, physician engineer	0.76(0.13, 4.23)	0.75
Self-employed	1.22(0.36, 4.13)	0.74
Unemployed	1.69(0.92, 3.07)	0.09
Retired	0.61(0.09,4.28)	0.62
Others	1.85(0.86, 3.96)	0.11
	14.18(0.90, 22.5)	0.06
**Mother’s occupation**		
Homemaker		
Worker	9.16(0.31, 1.47)	1.00
Employee	0.68(0.31, 1.47)	0.32
Faculty member, physician engineer	10.11 (0.49, 26.09)	0.14
Self-employed	0.71(0.03, 15.10)	0.83
Unemployed	4.28(1.36, 13.44)	0.01
Retired	0.24(0.02, 3.26)	0.28
	0.56(0.08, 4.01)	0.56
**Number of household members**	2.08(1.45, 2.99)	<0.001
**Ranking of child in the family**		
First		
Second	0.73(0.45, 1.17)	0.19
Third	0.40 (0.22, 0.74)	0.004
≥Fourth	0.36(0.18,0.74)	0.005
**Sex**		
Girl		
Boy	2.86(1.98,4.14)	<0.001

**Table 2:** The children’s physical activity level according to parents’ education

Years of education	Father/Mother	Children with low physical activity(%)	Children with moderate physical activity(%)	Children with vigorous physical activity (%)
Less than	Fathers	15.4	15.8	22.0
2 years	Mothers	24	18.2	35.4
2-11 years	Fathers	24.9	21.9	26.6
	Mothers	17.5	25.7	26.3
	Fathers	36.3	37.7	29.9
12 years*	Mothers	34.0	39.2	24.6
More than	Fathers	23.4	24.7	21.5
12 years	Mothers	14.5	16.9	13.7

* High school diploma

p<0.05 for all comparisons

**Table 3:** Correlation of social-cognitive variables with different physical activity levels in children and adolescents

	Low physical activity	Moderate physical activity	Vigorous physical activity level
Attitude	-0.233**	0.079	0.011
Subjective norm	-0.284**	-0.061	0.027
Perceived behavioral control	-0.159*	0.097	0.197*
Self efficacy	-0.205**	0.178*	0.009

* P value<0.05

** P value <0.01

## DISCUSSION

This study, which to the best of our knowledge is the first of its kind not only in Iran also in the Middle Eastern countries, revealed the main barriers of physical activity among a population-based sample of children and adolescents. Understanding these barriers might complement to existing Western literature by providing evidence from a population of children and adolescents with marked differences in socio-cultural and socio-cognitive context, and help to provide evidence-based preventive strategies, and to design appropriate interventions. Lack of support of families for promoting physical activity, neither in being a proper role model for their children, nor in accompanying their children and providing psychological support were of important barriers for participating in physical activity.

According to parents, believing in children’s educational success as a priority and limited availability of safe and easyaccess places for activity were among the main inhibitors of children and adolescents’ active lifestyle. The lower physical activity level in those children and adolescents with higher socio-economic situation, who may have better access to physical activity facilities, might be an indicator of the greater importance of the parents’ belief on the priority of studying. However, as mentioned by school staff, insufficient public knowledge on how to integrate activity in the routine daily life might have a pivotal role in this context. Increasing the community knowledge about adopting physical activity habits in daily routine, and informing the families and students about the benefits of regular physical activity for improving learning and school performance would reduce the main barrier of the constraint of studying and being active.

The prevalence of sedentary and physical exercise behavior varies across countries and by gender. Boys exhibited more typical recreational sedentary behaviors but also were more physically active than girls.^23^–^27^ The results of the current study were in accord with these findings, hence designing physical activity promoting interventions especially in girls is highly recommended. Using the school facilities in non-school hours can be a feasible and culturally appropriate intervention for providing safe and easy-access place for increasing the activity of children and their families, especially for females with limited access to sport facilities. Some studies found that higher parental education levels are associated with higher physical activity levels among their children.^28^–^30^ None-theless, our findings were not consistent with these studies; this might be because of the sedentary lifestyle of higher-educated families in our community, in terms of sedentary leisure-time activities as well as low transportation activities. Furthermore, the focus of higher-educated families on better school performance of their children, and considering the parents’ high expectations on school performance of their children and the remarkable extent of effort put in this regard in Iranian families are considered as other reasons for this discrepancy between our findings with those of studies conducted in Western populations.

Among social-cognitive variables studied in the current study, the role of self-efficacy was apparent in different physical activity levels. The self-efficacy theory proposes that confidence in personal ability to consider a behavior influences the direction, intensity, and persistence of behavior.^1^,^31^ Those children and adolescents who had high self-efficacy about physical activity perceived fewer barriers to their physical activities or were less-influenced by these barriers. It is suggested that those individuals with high self-efficacy are more likely to act on their expectations of desirable outcomes of being physically active and are more likely to enjoy physical activity.^1^ This role among samples of adolescent girls and boys has been exhibited in some cross-sectional^32^,^33^ and longitudinal studies.^34^,^35^ Designed interventions to increase physical activity among this age group should target such socio-cognitive aspects.

Some limitations of the present study should be addressed. Similar to other questionnaire-based studies, the problems of underestimation or overestimation of the time spent on physical activity and the recall bias should be taken into account. The cross-sectional nature of the survey limits the interpretation of the associations demonstrated in this study. The main strengths of this study were its novelty in a non-Western population-based sample of children and adolescents, its mixed design of qualitative and quantitative parts and its diversity in sampling from various parts of urban and rural areas with different socio-demographic characteristics.

## CONCLUSION

This study explored the main individual and environmental barriers which influence the physical activity in Iranian children and adolescents. The influence of priority of studying and the family expectations on their children’s school performance, as well as lack of safe and easy-access place for physical activity were the main barriers. Increasing the public knowledge about adopting physical activity habits in daily routine, and informing the families and students about the benefits of physical activity to improve learning and school performance, as well as using the school facilities in non-school hours would reduce these main barriers, and should be considered in planning effective preventive strategies, and implementing appropriate interventions. Such policies should consider socio-cognitive issues especially the self-efficacy of children and adolescents.
